# Liver Fat Content Is Associated with Elevated Serum Uric Acid in the Chinese Middle-Aged and Elderly Populations: Shanghai Changfeng Study

**DOI:** 10.1371/journal.pone.0140379

**Published:** 2015-10-16

**Authors:** Huandong Lin, Qian Li, Xiaojing Liu, Hui Ma, Mingfeng Xia, Dan Wang, Xiaoming Li, Jiong Wu, Naiqing Zhao, Baishen Pan, Xin Gao

**Affiliations:** 1 Department of Endocrinology and Metabolism, Zhongshan Hospital, Fudan University, Shanghai, China; 2 Research center on aging and medicine, Fudan University, Shanghai, China; 3 Department of Geriatrics, Zhongshan Hospital, Fudan University, Shanghai, China; 4 Department of Laboratory Medicine, Zhongshan Hospital, Fudan University, Shanghai, China; 5 Department of Biostatistics, School of Public Health, Fudan University, Shanghai, China; Inserm, U1052, UMR 5286, FRANCE

## Abstract

**Background and Aims:**

Although many studies have indicated a relationship between nonalcoholic fatty liver disease (NAFLD) and hyperuricemia, a few studies specifically examining the effects of the severity of liver fat content (LFC) on serum uric acid (SUA) and the presence of hyperuricemia because of the limitation of the examination methods for NAFLD. In this study, we investigate the relationship between the NAFLD and SUA levels in the Chinese population using standardized quantitative ultrasound.

**Methods:**

A community-based study was conducted from May 2010 to December 2012. A total of 4,305 people aged 45 years and above without excessive drinking were enrolled. A standard interview and anthropometric and laboratory blood parameters were collected for each person. The standardized ultrasound hepatic/renal ratio and hepatic attenuation rate was used to quantify LFC.

**Results:**

The prevalence of NAFLD and hyperuricemia was 33.1% and 17.1%, respectively. A total of 23.5% of the NAFLD subjects had hyperuricemia, and their SUA was higher than that of non-NAFLD subjects (327.2±76.8 vs 301.9±77.4 μmol/L, P<0.001). The LFC was positively correlated with SUA (r = 0.130, P<0.001) and an independent factor for SUA (standardized β = 0.054, P<0.001). The OR for the presence of hypreuricemia was 1.175 (95% CI 1.048–1.318; P<0.001) with a 1 SD increase in the log LFC. LFC greater than 10% was related to elevated SUA and an increased presence of hyperuricemia.

**Conclusions:**

LFC accumulation was associated with an increase in the prevalence of hyperuricemia and elevated SUA in our community-based population. LFC greater than 10% is related to the risk for hyperuricemia.

## Introduction

Uric acid is the final end product of purine metabolism in humans. It is well known that hyperuricemia is a causative factor of gout. However, in recent years, there has been a renewed interest in hyperuricemia because of its association with a number of metabolic disorders other than gout e.g., obesity, hypertension, glucose intolerance, metabolic syndrome, atherosclerosis and cardiovascular disease [1,2,3,4,5,6].

Non-alcoholic fatty liver disease (NAFLD) is defined as the presence of a significant amount of fat accumulation in the liver and includes simple steatosis, non-alcoholic steatohepatisis (NASH) and cirrhosis. The worldwide prevalence of NAFLD in the general population is estimated to be approximately 20–30% in Western countries and 5–18% in Asia[7]. Recently, the prevalence of NAFLD has increased in China with the improvement of life conditions[8]. The association between NAFLD and serum uric acid level has been well documented. However, most studies have explored the impact of elevated serum uric acid on the risk of NAFLD occurrence because NAFLD is typically diagnosed by characteristic echo patterns upon ultrasonic examination, which is limited by interobserver and intraobserver variability[9] and poor sensitivity for detecting mild hepatic steatosis[10], and it is ultimately unable to provide an accurate measurement of LFC. Although some researchers have used liver biopsy to evaluate the impact of the severity of NAFLD on serum uric acid [11], because it is an invasive examination, it is limited for use in wide application in clinical practice. A standardized ultrasound hepatic/renal ratio and hepatic attenuation rate used to quantify LFC was established by our group [12] and has provided a manner to explore the impact of the accumulation of liver fat on serum uric acid in a large-scale population study.

Therefore, in this study, we investigated the relationship between NAFLD and the SUA level in the Chinese population and explored the effects of the severity of LFC on serum uric acid and the presence of hyperuricemia.

## Materials and Methods

### Study design and population

The participants in our study are from the Shanghai Changfeng Study, which took place from May 2010 to December 2012. The Shanghai Changfeng Study, which was approved by the Ethical Committee of Shanghai Zhongshan Hospital, Fudan University and was conducted in accordance with the guidelines of Declaration of Helsinki, is a community-based prospective cohort study of multiple chronic non-infectious diseases in middle-aged and elderly people [13]. Written informed consent was obtained from all study subjects whose deserved rights and interests were monitored by the ethical committee. A total of 5,103 consecutive participants (2,151 men and 2,952 women) aged 45 years and above were enrolled in our study. We excluded 798 subjects (707 men and 91 women) because of excessive drinking (over 140 g for males and over 70 g for females each week). As a result, 4,305 subjects (1,444 men and 2861 women) were included in the final analysis.

### Data Collection

Letters were sent to participants with instructions asking them to not alter their diet or physical activity for at least 3 days prior to examination. On the examination day, a questionnaire was administered by a trained researcher to collect information for each participant regarding their lifestyle and medical history. Then, the body weight, height, waist circumference (WC, midway between the lowest rib margin and iliac crest) and hip circumference (HC, widest level over the greater trochanters) of each participant clothed in a light gown was measured. The body mass index (BMI) was calculated as the weight divided by height squared (kg/m^2^). The waist-to-hip ratio (WHR) was calculated as the waist circumference divided by the hip circumference. The resting blood pressure (BP) was measured three times with an electronic blood pressure monitor (OMRON Model HEM-752 FUZZY, Omron Co., Dalian, China), and then the average was calculated.

Blood samples were collected after a fasting period of at least 10 hours overnight. Fasting blood glucose (FBG), total cholesterol (TC), triglycerides (TGs), high-density lipoprotein-cholesterol (HDL-C), low-density lipoprotein-cholesterol (LDL-C), alanine aminotransferase (ALT), aspartate aminotransferase (AST), γ-glutamyltransferase (GGT), alkaline phosphatase (ALP), blood urea (BU), creatinine (Cr), and uric acid (UA) were measured with an automated bio-analyzer (HITACHI 7600, Tokyo, Japan). The eGFR was calculated using the Modification of Diet in Renal Disease (MDRD) study formula[14] as follows: 186×[serum creatinine (mg/dl)]^-1.154^×(age)^-0.203^×[0.742 (if female)]. The 2-hour blood glucose (2hBG) was measured after a 75 g oral glucose tolerance test, and HbA_1C_ was measured by high performance liquid chromatography (HPLC) (BIO-RAD II TURBO), which was standardized to the National Glycated Haemoglobin Standardization Program (NGSP).

Hepatic ultrasonography scanning was performed on all subjects by an experienced radiologist who was blinded to the subjects’ medical information using a GE LOGIQ P5 scanner (GE Healthcare, Milwaukee, USA) with a 4-MHz probe. The interobserver agreement for US hepatic/renal ratio and US hepatic attenuation rate was excellent (ICC = 0.956 and ICC = 0.942, respectively). We measured LFC according to the procedure and formula described elsewhere [12], and we used 9.15% as a cut-off value for diagnosing liver steatosis. Hyperuricemia was defined as a serum UA level > = 420 μmol/L in men and > = 360 μmol/L in women.

### Statistical analysis

All statistical analyses were performed using SPSS software version 16.0 (SPSS, Chicago, IL, USA). Continuous variables were presented as the means±SD with the exception of skewed variables, which were presented as medians with the interquartile range (25–75%) provided in parentheses.

The one-way analysis of variance t test or Mann-Whitney U test was used for comparisons of continuous data among groups, whereas the Chi-squared test was used for comparisons of categorical variables. Pearson or spearman analyses were used to determine the association between serum UA level and other clinical parameters (logarithmic transformed for skewed variables, expressed as LG). Stepwise logistic regression analysis (forward; Wald) was used to evaluate independent risk factors for hyperuricemia. Multiple linear regression analysis was used to determine the association between the serum UA level and other parameters. All statistical tests were two tailed, and p-values less than 0.05 were considered significant.

## Results

### Characteristics of the study participants

The characteristics of the participants are shown in Table 1. In our study population, the prevalence of NAFLD and hyperuricemia was 33.1% and 17.1%, respectively. The prevalence of hyperuricemia was higher in patients with NAFLD than that in those without NAFLD (23.5% vs 13.9%, P<0.001). Patients with NAFLD were younger, had heavier body weights, and higher blood pressures and glucose levels than those without NAFLD. The total cholesterol, triglyceride and transaminase levels were higher, whereas HDL-C was lower in NAFLD patients than in those without NAFLD. Moreover, the level of serum UA was higher in NAFLD than in non-NAFLD participants (327.2±76.8 vs 301.9±77.4 μmol/L, P<0.001).

**Table 1:** Clinical characteristics of the participants.

	All (n = 4,305)		Male (n = 1,444)		Female (n = 2,861)	
	Non-NAFLD	NAFLD	Non-NAFLD	NAFLD	Non-NAFLD	NAFLD
n (%)	2,881 (66.9)	1,424 (33.1)	992 (68.7)	452 (31.3)	1,889 (66.0)	972 (34.0)
Age (y)	64.0±9.9	62.7±9.0***	66.0±9.8	63.2±9.2***	62.9±9.7	62.5±8.8
BMI (kg/m^2^)	23.3±3.1	25.6±3.2***	23.6±3.0	25.8±2.8***	23.1±3.2	25.6±3.4***
WC (cm)	81.3±9.3	87.6±9.0***	84.2±8.7	91.3±8.3***	79.7±9.2	85.8±8.9***
WHR	0.89±0.08	0.92±0.07***	0.92±0.09	0.95±0.06***	0.88±0.08	0.90±0.07***
SBP (mmHg)	132.8±19.4	136.8±18.7***	134.7±18.4	136.7±17.7	131.9±19.8	136.8±19.2***
DBP (mmHg)	74.1±9.8	77.4±10.0***	76.0±10.2	79.3±9.6***	73.1±9.4	76.6±10.1***
TC (mmol/L)	5.06±0.91	5.19±0.98***	4.68±0.85	4.83±0.89**	5.26±0.87	5.36±0.97*
TG (mmol/L)	1.50±0.91	2.16±1.62***	1.52±0.98	2.09±1.39***	1.49±0.87	2.19±1.71***
HDL-C (mmol/L)	1.50±0.39	1.35±0.33***	1.32±0.32	1.19±0.27***	1.56±0.39	1.42±0.33***
LDL-C (mmol/L)	2.89±0.78	2.92±0.84	2.69±0.73	2.73±0.78	3.00±0.78	3.00±0.85
FBG (mmol/L)	5.42±1.29	5.89±1.70***	5.58±1.57	6.00±1.75***	5.34±1.10	5.83±1.67***
2hBG (mmol/L)	7.14±3.00	8.64±3.58***	7.41±3.07	8.99±3.76***	7.01±2.97	8.47±3.49***
HbA_1C_ (%)	5.77±0.81	6.07±1.02***	5.85±1.00	6.11±1.11***	5.73±0.68	6.05±0.98***
ALT (U/L)	15 (11–19)	18 (14–26)***	16 (12–21)	20 (15–29) *	14 (11–19)	18(13–25)***
AST (U/L)	20 (17–23)	21 (18–25)***	20 (17–23)	21 (18–25)***	20 (17–23)	21 (18–24)***
GGT (U/L)	20 (16–28)	26 (20–38)***	23 (18–32)	31 (22–41) ***	19 (15–26)	24 (19–35)***
ALP (U/L)	71 (61–84)	73 (62–87) ***	69 (59–81)	71 (59–84) *	72 (61–85)	75 (63–89) **
LFC (%)	3.4(1.4–5.6)	14.8 (11.7–18.7)***	3.1(1.1–5.2)	14.8 (11.6–19.4)**	3.6(1.5–5.8)	14.8 (11.7–18.5)***
BU (mmol/L)	5.4±1.5	5.3±1.3	5.7±1.5	5.5±1.3	5.3±1.4	5.2±1.3
Cr (umol/L)	68.9±21.5	66.5±16.9***	82.7±20.4	80.6±16.3	61.7±18.3	60.0±12.5***
UA (umol/L)	301.9±77.4	327.2±76.8***	342.8±79.7	362.5±75.2***	280.5±66.8	310.8±71.9***
eGFR (ml/min 1.73 m^2^)	93.4±19.9	96.1±20.0***	90.3±19.2	93.1±19.1*	95.0±20.0	97.4±20.2**
Hyperuricemia (%)	13.9	23.5***	17.2	23.9**	12.2	23.3***
Drugs-taking^§^ (%)	0.4%	0.6%	1.1%	1.5%	0.1%	0.1%

Data are expressed as the means±SE, percentages or medians (25^th^ to 75^th^ percentiles). Compared with Non-NAFLD group

*: P<0.05

**: P<0.01

***: P<0.001.

NAFLD: nonalcoholic fatty liver disease; BMI: body mass index; WC: waist circumference; WHR: waist-to-hip ratio; SBP: systolic blood pressure; DBP: diastolic blood pressure; TC: total cholesterol; TG: triglycerides; HDL-C: high-density lipoprotein-cholesterol; LDL-C: low-density lipoprotein-cholesterol; FBG: fasting blood glucose; 2hBG: 2-hour blood glucose; ALT: alanine aminotransferase; AST: aspartate aminotransferase; GGT: γ-glutamyltransferase; ALP: alkaline phosphatase; LFC: liver fat content; BU: blood urea; UA: uric acid; eGFR: estimated glomerular filtration rate.

§: Rate of drugs-taking for hyperuricemia or gout.

### Relationship between LFC and level of serum uric acid

Univariate correlation analysis demonstrated that the level of serum uric acid significantly and positively correlated with age, BMI, WC, WHR, BP, TG, FBG, 2hBG, ALT, AST, GGT, LFC, BU and Cr, and it significantly and negatively correlated with HDL-C and eGFR (Table 2). Multiple stepwise regression analysis showed that LFC was an independent factor of the SUA level (Table 3). In addition, the SUA concentration increased with increases in LFC. When the LFC was greater than 10%, the SUA was significantly higher than LFC <5% (Table 4).

**Table 2:** Correlation coefficients between serum uric acid and all related clinical parameters, including LFC.

	Total (n = 4305)		Male (n = 1444)		Female (n = 2861)	
	*r*	*P*	*r*	*P*	*r*	*P*
Age	0.175	<0.001	0.084	0.001	0.178	<0.001
BMI	0.269	<0.001	0.215	<0.001	0.299	<0.001
WC	0.324	<0.001	0.198	<0.001	0.308	<0.001
WHR	0.248	<0.001	0.090	0.001	0.237	<0.001
SBP	0.197	<0.001	0.144	<0.001	0.222	<0.001
DBP	0.147	<0.001	0.114	<0.001	0.108	<0.001
TC	-0.022	0.148	0.086	0.001	0.091	<0.001
TG	0.204	<0.001	0.206	<0.001	0.232	<0.001
HDL-C	-0.291	<0.001	-0.162	<0.001	-0.222	<0.001
LDL-C	-0.013	0.383	0.032	0.225	0.065	0.001
FBG	0.033	0.033	-0.132	<0.001	0.109	<0.001
2hBG	0.157	<0.001	0.031	0.277	0.213	<0.001
HbA_1C_	0.026	0.106	-0.156	<0.001	0.131	<0.001
LG_ALT	0.174	<0.001	0.141	<0.001	0.157	<0.001
LG_AST	0.124	<0.001	0.139	<0.001	0.127	<0.001
LG_ALP	-0.007	0.658	-0.011	0.669	0.046	0.014
LG_GGT	0.256	<0.001	0.204	<0.001	0.227	<0.001
LG_LFC	0.130	<0.001	0.107	<0.001	0.192	<0.001
BU	0.251	<0.001	0.188	<0.001	0.250	<0.001
Cr	0.423	<0.001	0.350	<0.001	0.278	<0.001
eGFR	-0.363	<0.001	-0.382	<0.001	-0.332	<0.001

LFC: liver fat content; BMI: body mass index; WC: waist circumference; WHR: waist-to-hip ratio; SBP: systolic blood pressure; DBP: diastolic blood pressure; TC: total cholesterol; TG: triglycerides; HDL-C: high-density lipoprotein-cholesterol; LDL-C: low-density lipoprotein-cholesterol; FBG: fasting blood glucose; 2hBG: 2-hour blood glucose; LG: logarithmic transformed; ALT: alanine aminotransferase; AST: aspartate aminotransferase; GGT: γ-glutamyltransferase; ALP: alkaline phosphatase; BU: blood urea; eGFR: estimated glomerular filtration rate.

**Table 3:** Factors associated with the level of serum uric acid.

	β	95% CI	SE	Standardized β	*P*
Cr	1.761	1.625~1.897	0.069	0.384	<0.001
WC	0.942	0.578~1.307	0.186	0.116	<0.001
LG_GGT	37.187	26.706~47.669	5.346	0.111	<0.001
TG	5.289	3.385~7.193	0.971	0.083	<0.001
BU	4.736	3.050~6.422	0.860	0.080	<0.001
HDL-C	-17.221	-23.620~-10.821	3.264	-0.083	<0.001
SBP	0.274	0.157~0.391	0.060	0.066	<0.001
LG_LFC	9.156	4.333~13.978	2.460	0.054	<0.001
BMI	1.459	0.423~2.496	0.528	0.062	0.006
LG_AST	25.210	6.229~44.191	9.681	0.039	0.009
FPG	-3.809	-6.368~-1.251	1.305	-0.049	0.004
2hBG	0.923	0.113~1.733	0.413	0.039	0.025

Dependent variable: serum uric acid. Independent variables: age, BMI, WC, WHR, SBP, DBP, FBG, 2hBG, LG_ALT, LG_AST, LG_GGT, TG, HDL-C, BU, Cr, eGFR and LG_LFC. Adjusted for drugs-taking for hyperuricemia or gout.

Cr: creainine; WC: waist circumference; LG: logarithmic transformed; GGT: γ-glutamyltransferase; TG: triglycerides; BU: blood urea; HDL-C: high-density lipoprotein-cholesterol; SBP: systolic blood pressure; LFC: liver fat content; BMI: body mass index; AST: aspartate aminotransferase; FBG: fasting blood glucose; 2hBG: 2-hour blood glucose; BMI: body mass index; WHR: waist-to-hip ratio; DBP: diastolic blood pressure; ALT: alanine aminotransferase; eGFR: estimated glomerular filtration rate.

**Table 4:** Levels of serum uric acid with increases in LFC.

LFC	Total (n = 4,305)		Male (n = 1,444)		Female (n = 2,861)	
	n	UA (μmol/L)	n	UA (μmol/L)	n	UA (μmol/L)
<5%	2003	301.4±78.3	719	340.5±80.4	1284	279.5±67.8
5~10%	993	303.2±75.1	310	347.4±77.1	683	283.1±64.9
10~15%	628	326.0±77.5*	196	364.9±75.2*	432	308.4±72.0*
15~20%	406	329.6±77.1*	119	366.6±75.9*	287	314.3±72.3*
> = 20%	275	336.3±73.9*	100	361.8±74.6*	175	321.7±69.7*
*P*		<0.001		<0.001		<0.001

LFC: liver fat content; UA: serum uric acid

*: compared with LFC<5%, P<0.05.

### Relationship between LFC and hyperuricemia

**Fig 1:**
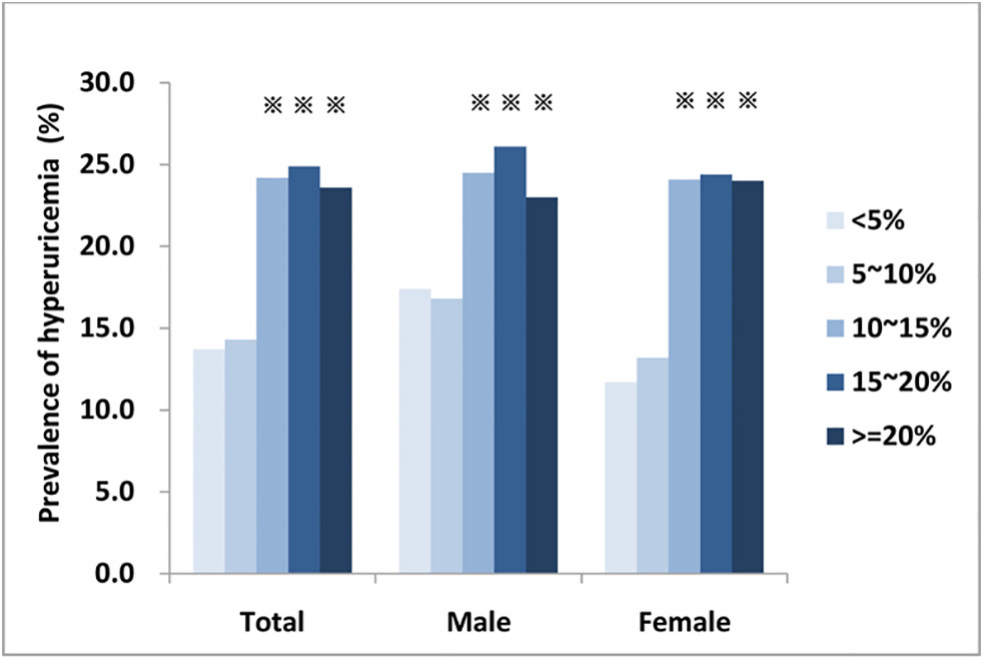
Prevalence of hyperuricemia with increases in the LFC. In total, the prevalence of hyperuricemia was 13.7%, 14.3%, 24.2%, 24.9% and 23.6%, respectively with each additional 5% of stratified LFC from <5% to > = 20%. In male, the prevalence was 17.4%, 16.8%, 24.5%, 26.1% and 23.0%, respectively; in female, the prevalence was 11.7%, 13.2%, 24.1%, 24.4% and 24.0%, respectively. ※: compared with LFC<5%, *P*<0.05

**Fig 2:**
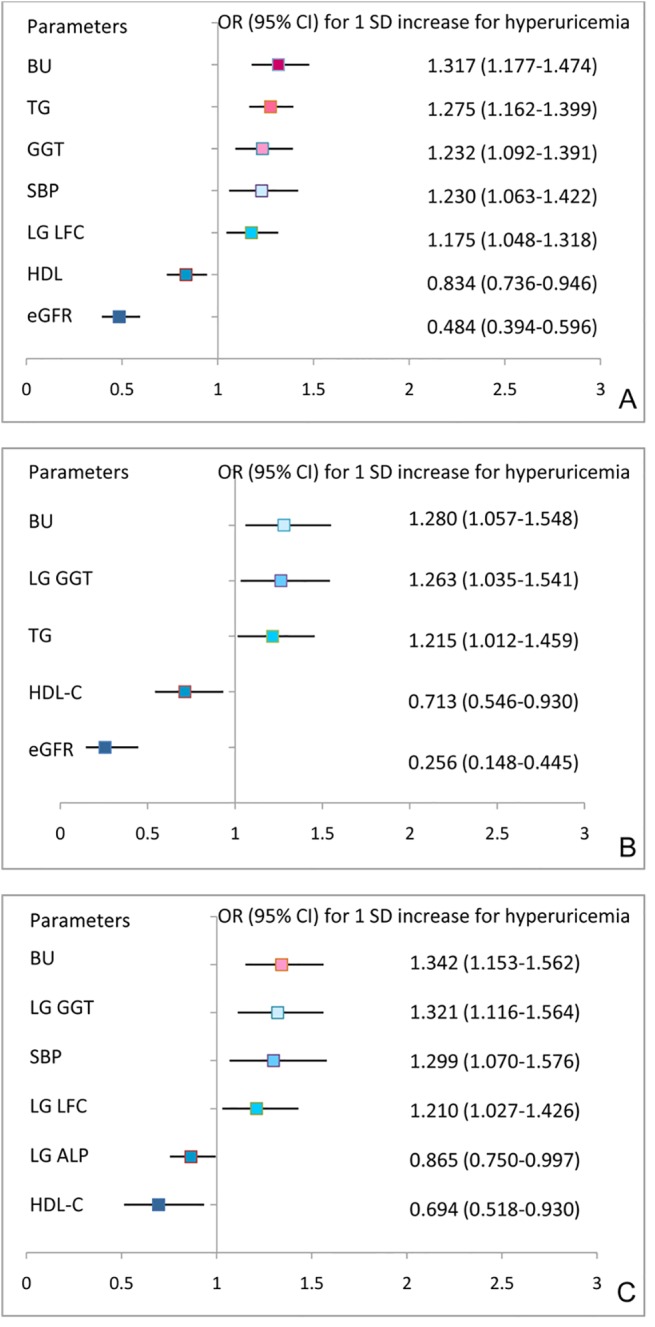
ORs for 1 SD increases in variables with the prevalence of hyperuricemia from multiple logistic regression analysis. (A) The total study population, including the variables gender, age, BMI, WC, WHR, SBP, DBP, TG, HDL-C, FPG, 2hPG, ALT, AST, GGT, LFC, BU, Cr, and eGFR, which were significantly correlated with serum uric acid in bivariate correlate analysis. (B) The male subset, including the variables age, BMI, WC, WHR, SBP, DBP, TC, TG, HDL-C, FPG, HbA_1C_, ALT, AST, GGT, LFC, BU, Cr and eGFR, which were significantly correlated with serum uric acid in bivariate correlation analysis. (C) The female subset, including the variables age, BMI, WC, WHR, SBP, DBP, TC, TG, HDL-C, LDL-C, FPG, 2hPG, HbA_1C_, ALT, AST, ALP, GGT, LFC, BU, Cr and eGFR, which were significantly correlated with serum uric acid in bivariate correlation analysis.

The prevalence of hyperuricemia increased with each additional 5% of stratified LFC (Fig 1). There was no significant difference between LFC <5% and LFC 5~10%, but the prevalence of hyperuricemia significantly increased when the LFC was greater than 10% compared with LFC<5%, and there was no difference in this correlation for either males of females. Compared with the LFC<5% group, the OR (95% CI) for the presence of hyperuricemia associated with the LFC 10~15%, 15~20% and greater than 20% groups was 1.138 (1.085–1.194), 1.148 (1.083–1.218) and 1.130 (1.055–1.209), respectively, for the total population, and 1.163 (1.099–1.231), 1.168 (1.091–1.251) and 1.162 (1.067–1.266), respectively, for females alone (Table 5). Multiple logistic regression analysis demonstrated that LFC was an independent factor for hyperuricemia in the total and female population. For a 1 SD increase in the Log LFC, the risk for the occurrence of hyperuricemia was 1.175 (95% CI 1.048–1.318) for the total population and 1.210 (95% CI 1.027–1.426) for females alone. (Fig 2).

**Table 5:** ORs for hyperuricemia with 5% increases in the LFC.

	Total (n = 4,305)		Male (n = 1,444)		Female (n = 2,861)	
	OR	95%CI	OR	95%CI	OR	95%CI
<5%	1.000		1.000		1.000	
5~10%	1.007	0.976–1.038	0.993	0.935–1.054	1.017	0.982–1.054
10~15%	1.138	1.085–1.194	1.094	1.003–1.193	1.163	1.099–1.231
15~20%	1.148	1.083–1.218	1.117	0.999–1.249	1.168	1.091–1.251
> = 20%	1.130	1.055–1.209	1.073	0.959–1.200	1.162	1.067–1.266

LFC: liver fat content; OR: odds ratio; CI: confidence interval.

## Discussion

In our study, we found that 23.5% of people with NAFLD had hyperuricemia; LFC was an independent factor of hyperuricemia and positively correlated with serum uric acid for both men and women. The prevalence of hyperuricemia and serum uric acid increased with increases in LFC, and a LFC greater than 10% had a high risk for the occurrence of hyperuricemia.

The prevalence of hyperuricemia in the general population was 8.38~14.71% [15,16]. However, the prevalence of hyperuricemia in patients with NAFLD was 20~33.4% [11,17,18], which was higher than that of the general population. We also found that 23.5% of people with NAFLD had hyperuricemia. Our results are similar to those of previous studies and indicate that hepatic steatosis has a close relationship with elevated serum uric acid. However, these previous studies have used conventional ultrasound [16], which cannot relate the severity of hepatic steatosis to the level of serum uric acid or biopsy [11,17], an invasive examination, which cannot be widely used for clinical diagnoses or therapeutic follow-up. Thus, our study using semi-quantitative ultrasonography try to determine the LFC, as the severity of NAFLD, with hyperuricemia or serum uric acid.

In our study, we found that serum uric acid was positively correlated with LFC, and there was no difference in this correlation for either males or females. Although the risk of the presence of hyperuricemia increased with a 1 SD increase in log LFC, multiple regression analysis confirmed that LFC was an independent factor for serum uric acid level but for only female subjects. Moreover, we found that a mild increase in LFC (less than 10%) did not increase serum uric acid and the presence of hyperuricemia. However, when the LFC was greater than 10%, there was a significant increase despite the level of serum uric acid or the presence of hyperuricemia. Furthermore, there was no further increase in serum uric acid and presence of hyperuricemia with increased LFC. These results suggest that the LFC exceed 10% might be the threshold for the presence of hyperuricemia. Our previous studies indicated that the early phase of beta-cell function was deteriorated as the LFC accumulated to 10% [19,20], and the participants with LFC higher than 10% had higher odds ratios of impaired glucose regulation as compared with those with LFC below 10% after adjustment for all confounding risk factors [21]. So hyperinsulinaemia or insulin resistance induced by beta-cell dysfunction might increase serum uric acid through increased uric acid production and decreased renal excretion of uric acid [22]. Furthermore, considering the dual role of uric acid as an anti-oxidant and pro-oxidant[23], we speculate that uric acid may change its role from an anti-oxidant to a pro-oxidant when the LFC reaches 10%.

NAFLD, including simple steatosis, NASH and cirrhosis[24], is the result of hepatic fat accumulation in patients without a history of excessive alcohol consumption[25]. Recent studies have indicated that NAFLD is linked to increased risk for cardiovascular disease[26]. Similarly, hyperuricemia, previously considered the cause for gout, was also recently found to be linked to cardiovascular disease [4]. After the first case-controlled study describing an association between NAFLD and serum uric acid by Lonardo et al. [27], several studies have reported a close relationship between NAFLD and uric acid [11,15,16,17,18]. However, the mechanisms by which NAFLD associates with serum uric acid remain unclear. Most studies have considered that the increased prevalence of NAFLD is due to elevated serum uric acid[16]. Elevated serum uric acid at baseline increases the risk for NAFLD [28,29]. However, some studies have indicated that the accumulation of hepatic fat or steatohepatitis induces increased serum uric acid [11,30]. Hyperinsulinemia or insulin resistance induced by hepatic steatosis increases the production of uric acid, reduces uric acid excretion [3,22] and eventually results in hyperuricemia. Elevated serum uric acid may be a marker of endogenous inflammatory cytokines responding to hepatocyte damage[31].

In conclusion, the clinical implications of our study are as follows: 1) we found that the accumulation of LFC is associated with an increased prevalence of hyperuricemia and elevated serum uric acid in the general population, and 2) there is a threshold value for the LFC associated with the risk of hyperuricemia or elevated serum uric acid. We should be concerned with people who have greater than 10% LFC and metabolic abnormalities. The limitations of our study are as follows: 1) this was a study of the middle-aged and elderly population, we could not select gold standard, such as liver biopsy or magnetic resonance spectroscopy, for diagnosing NAFLD and we also could not distinguished patients with NASH and non-NASH which required to be diagnosed by liver biopsy as gold standard; 2) we could not consider the role of insulin resistance in the relationship between NAFLD and hyperuricemia; and 3) our study was a cross-section study, which could not demonstrate a clear causal relationship between NAFLD and hyperuricemia. Further prospective studies are needed to eliminate the causal relationship with these two diseases.

## Abbreviations

NAFLD: Nonalcoholic fatty liver disease
LFC: Liver fat content
SUA: serum uric acid
NASH: Non-alcoholic steatohepatisis
WC: waist circumference
HC: Hip circumference
BMI: Body mass index
WHR: Waist-to-hip ratio
BP: Blood pressure
FBG: Fasting blood glucose
TC: Total cholesterol
TGs: Triglycerides
HDL-C: High-density lipoprotein-cholesterol
LDL-C: Low-density lipoprotein-cholesterol
ALT: Alanine aminotransferase
AST: Aspartate aminotransferase
GGT: γ-glutamyltransferase
ALP: Alkaline phosphatase
BU: Blood urea
Cr: Creatinine
UA: Uric acid
MDRD: Modification of Diet in Renal Disease
2hBG: 2-hour blood glucose
HPLC: High performance liquid chromatography
NGSP: National Glycated Haemoglobin Standardization Program
